# New Diagnostic and Therapeutic Targets for Spinal Cord Injury: GRN Gene

**DOI:** 10.1111/jcmm.70749

**Published:** 2025-07-30

**Authors:** Han Ding, Lei Feng, Jianping Zhang, Tuo Fang, Jun Shang, Ke Fang, Shiqing Feng

**Affiliations:** ^1^ Tianjin Key Laboratory of Spine and Spinal Cord, National Spinal Cord Injury International Cooperation Base, Department of Orthopedics Tianjin Medical University General Hospital Tianjin China; ^2^ Orthopaedic Bioengineering Research Group, Division of Surgery and Interventional Science University College London London UK; ^3^ Institute of Medical Sciences, The Second Hospital and Orthopedic Research Center of Shandong University, Cheeloo College of Medicine Shandong University Jinan Shandong China

**Keywords:** biomarkers, granulin, immune cell infiltration, m6A methylation, spinal cord injury

## Abstract

Spinal cord injury (SCI) is a severe disabling disease due to the poor self‐healing of the central nervous system. Studies showed that many N6‐methyladenosine (m6A) RNA methylation profiles are hypomethylated after SCI, which are related to neural regeneration and different m6A marker genes. In addition, immune cell infiltration may significantly affect the development and progression of SCI. Therefore, we attempted to identify the correlation between SCI‐related biomarkers and m6A methylation regulators in order to classify them. To this end, we collected two gene expression profile datasets (GSE464 and GSE45006) from the GEO database, performed differential expression analysis between pairs before and after SCI, and identified 19 constant differentially expressed genes (DEGs). We found that the constant differential genes were strongly correlated with m6A methylation regulators, which could modulate the immune microenvironment of SCI. Next, this paper used a consensus clustering algorithm to classify SCI patients into three subtypes. There are significant differences between 19 constant DEGs and 28 immune cells among different subtypes. Finally, the correlation analysis of the intersection genes between constant DEGs and immune genes was performed, and GRN was identified as a potential immune biomarker for SCI.

## 1 Introduction

Spinal cord injury (SCI) is a common disabling disease that may lead to neurological damage and dysfunction [1, 2]. At present, there is a lack of effective therapeutic intervention for SCI. Most treatment options focus on stabilising the condition and preventing further damage [3]. Currently, the effective treatment for SCI is limited, and the mechanism of its occurrence and development is still unclear. Therefore, it is necessary to develop new methods for the treatment of SCI from the perspective of molecular mechanisms.

N6‐methyladenosine (m6A) methylation is a common modification of messenger RNA (mRNA) in eukaryotes [4]. Such modification was proven to affect many biological processes and plays an essential role in mRNA splicing, export, translation, and other processes [5]. In addition, m6A methylation regulators were shown to have both pro‐nd anti‐tumour effects during the initiation and progression of various tumours. Guo et al. constructed the prognostic features of oesophageal squamous cell carcinoma based on m6A RNA methylation regulators and explored the potential association among these regulators and the tumour immune microenvironment [6]. Zhang et al. [7] found that m6A‐related genes METTL3, YTHDF1, and YTHDF2 can be identified as novel biomarkers for the prognosis of lung adenocarcinoma. Recent studies found that the level of m6A modification changes before and after the onset of SCI, and m6A‐related modulators are involved in the microenvironment after SCI [8]. For example, several studies found a significant increase in the content of m6A regulators (METTL3 and METTL14) in the lesion site after SCI [9, 10]. Recently, several studies have shown that immune infiltration plays a significant role in the development and treatment of spinal cord injury [11, 12]. N6‐methyladenosine (m6A) modification has attracted much more attention in evaluating immune infiltration and determining the composition of infiltrating immune cells [13, 14], especially in spinal cord injury [15]. However, the pathological changes of relevant nerve immune cells (especially microglia) and the biological processes involved in SCI after m6A modification must be further studied. In addition, there are differences in treatment methods and therapeutic effects among patients with different SCI, so it is necessary to explore the differences in the molecular mechanisms of different subtypes of SCI.

GRN, which is called the precursor gene of granular protein (Progranulin), encodes a secreted glycoprotein, which can be further cleaved to produce many active peptides with a molecular weight of 6 kDa. These peptides can regulate cell growth and play an important role in normal development, wound healing, tumourigenesis, and neurodegenerative diseases. GRN plays an important role in nervous system diseases, especially in neurodegenerative diseases such as Frontotemporal Lobar Degeneration (FTLD), Alzheimer's Disease (AD), Parkinson's Disease (PD) and Amyotrophic Lateral Sclerosis (ALS). Although there is no direct evidence that the expression of the GRN gene is directly regulated by m6A methylation, considering the extensive role of m6A methylation in regulating gene expression and cell function, it can be inferred that m6A methylation may indirectly affect the function of the GRN gene. In addition, the role of m6A methylation in neural development and neurodegenerative diseases indicates that it may overlap with the role of the GRN gene in nervous system diseases.

To this end, two rats SCI datasets (GSE464 and GSE45006) from the GEO database were collected, debatched, and merged. The combined dataset was grouped according to multiple time points before and after SCI, and constant DEGs were obtained by differential analysis. In detail, we analysed the correlation between 19 constant DEGs and m6A methylation regulators and the immune microenvironment. Finally, SCI was divided into three subtypes by consensus clustering method, and the differences in gene expression and microglia content among the three subtypes were discussed in detail. In addition, the intersection of GRN between constant DEGs and immune genes was identified as a potential immunobiological marker for SCI.

## 2 Methods

### 2.1 Download and Grouping of Expression Matrix Data

TCGA and GEO belong to public databases. The patients involved in the database have obtained ethical approval. Users can download relevant data for free for research and publish relevant articles. Our study is based on open‐source data, so there are no ethical issues and other conflicts of interest. We used two datasets GSE464 and GSE45006 from the GEO database (https://www.ncbi.nlm.nih.gov/geo/). For the GSE464 expression matrix, the SCI model was established by the weightlessness method around the T8‐9 segment of rats. The rats were then available at 1 day, 14 days, and week > 4 after laminectomy as a control spinal cord gene expression microarray. In this study, we performed gene annotation on the GSE45006 dataset based on the GPL85 platform. For the GSE45006 expression matrix, the thoracic spinal cord (T7) of rats was injured by aneurysm clipping, and the same grouping method was adopted as the GSE464 expression matrix. The GSE151371 expression matrix was used for the external test set of this paper. The data contained SCI blood RNA expression from 10 healthy controls, 10 non‐CNS trauma controls, and 38 SCI patients. In addition, gene symbol annotation of the GSE464, GSE45006, and GSE151371 expression matrices was performed based on GPL85, GPL1355, and GPL20301 platforms, respectively. Finally, this paper removed samples without gene symbols, and the multiple‐sample average of gene symbols was calculated.

### 2.2 Data Merged and Batch Normalised

In this study, the GSE464 and GSE45006 expression matrices were corrected respectively. The two expression matrices were merged, and the combined microarray data were background corrected and batch normalised using the “sva” R package [16]. The merged expression matrix contained the set of both data types for all time points.

### 2.3 Identification of Differentially Expressed Genes

In this study, the limma package in R [17] was used to perform differential expression analysis on the combined microarray datasets and set at *p* value less than 0.05 to obtain DEGs of different time points (1 day, 14 days and week > 4).

### 2.4 Enrichment Analysis of DEGs

In this study, the DEGs enriched pathways were analysed by cluster profile package of R software (adjusted *p* value < 0.05). Specifically, Gene Ontology (GO) enrichment analysis [18, 19] and Kyoto Encyclopedia of Genes and Genomes (KEGG) pathway enrichment analysis [20, 21] were performed for DEGs. This paper divided GO enrichment analysis into three categories of pathways: biological process (BP), cellular component (CC) and molecular function (MF).

### 2.5 Estimation of Immune Cell Infiltration

To quantify the relative abundance of cellular infiltration in spinal cord, we stored many subtypes of human immune cells, such as activated dendritic cells and CD8+ T cells according to the gene sets obtained by Charoentong et al., which labelled each infiltrating immune cell in the tumour microenvironment [22, 23]. Enrichment scores calculated for gene sets using the single‐sample Gene‐set enrichment analysis (ssGSEA) algorithm represent the relative abundance of infiltrating cells in each sample.

### 2.6 Unsupervised Clustering of 19 Constant DEGs

To identify the different molecular subtypes of SCI, 19 DEGs were identified in this paper for cluster analysis. Specifically, this paper used the consensus clustering algorithm to obtain the number of clusters and the corresponding stability [24]. To ensure the stability of clustering, this paper used the ConsensuClusterPlus package of R software to repeat the above steps 1000 times [25].

### 2.7 Rats Spinal Cord Injury Model

Female Wistar rats (7 weeks old) weighing 200 ± 10 g were used in this study (Vital River, Beijing, China). The rats were kept in the environment with a 12‐h light–dark cycle at the temperature of 20°C–25°C and humidity of 40%–60%, with free access to food and water. The animal study was reviewed and approved by the Ethics Committee of the Institute of Tianjin Medical University General Hospital (approval number: IRB2022‐DW‐45) and performed according to the national guidelines for laboratory animal use and care. The rats were anaesthetised with isoflurane (RWD, R510‐22, Guangdong, China). Laminectomy was performed at the T10 vertebrae and the spinal cord was fully exposed. The NYU Impactor device (W. M. Keck, USA) was used to establish the SCI model with a 10 g node at a height of 25 cm. After confirming that the contusion was successful, the muscles, fascia, and skin were sutured. The bladder was expressed twice a day after SCI manually. Cefuroxime sodium (Hongtu, Nanjing, China) was administered to prevent infection.

### 2.8 Magnetic Resonance Imaging (MRI)

The collection of routine MRI data for spinal cord injury includes the length of intramedullary lesions, diffusion rate of intramedullary lesions, axial diffusion rate, etc., and can collect relevant data for hemorrhagic and non‐hemorrhagic contusion lesions through SWI sequence. Even collect FA, MD, RD, AD and other data through DTI. The collection of rats magnetic resonance data is limited by the individual size of experimental animals and the characteristics of modelling, making it difficult to achieve the above data collection. Through repeated observation of the obtained MRI images of rats, the author chose to measure and collect data by extending a range of 2.5 mm towards the head and tail of the rat spinal cord as the centre. The magnetic resonance imaging of experimental rats was collected using a Philips model with a coil diameter of 1.0 mm. T2WI sequence was selected, with a repetition time of 2000 ms, an echo time of 65 ms, a flip angle of 90°, imaging pixels of 220 × 120, layer thickness of 2 mm and FOV of 120 × 80 mm. After shooting, download from the cloud workstation (ISP 9, Philips Healthcare, Best, the Netherlands). On the MRI sagittal position, the total observation range is 5 mm. After being divided into five equal parts, the diameter of the spinal cord is measured, and the circumference and spinal cord area within the specified range were calculated. We used RadiAnt DICOM Viewer (64bit) software for data analysis, unified the magnetic resonance images through the toolbar, selected Zoom image to expand by 800 times, selected Sharpen3 in Image filters to perform black and white contrast enhancement on the images and used closed polygon to calculate the circumference and area of the specified range.

### 2.9 Real‐Time Polymerase Chain Reaction

T10 spinal cords were collected 1 day after SCI or laminectomy, and frozen and stored at −80°C immediately. Total RNA was extracted using Trizol reagent (Cat# R0016; Beyotime Biotechnology). The HiFiScript gDNA Removal RT MasterMix kit (Cat# CW2020M; CWBio, Beijing, China) was used to synthesise cDNA. RT‐qPCR was performed with UltraSYBR One Step RT‐qPCR Kit (Cat# CW0659S; CWBio) and the reactions were performed in a LightCycler 96 instrument (Roche, Basel, Switzerland) in accordance with the manufacturer's instructions.

### 2.10 Statistical Analysis

This paper used the Spearman's correlation coefficient and distance correlation analysis to obtain the correlation coefficient between immune cells and constantly differentially expressed gene expression. In addition, one‐way ANOVA and the Kruskal‐Wallis test [26] were used in this paper to compare the differences between multiple samples. In the statistical analysis, the *p* value was two‐sided, and a *p* value of less than 0.05 was considered statistically significant.

## 3 Results

### 3.1 Normalisation and Batch Correction of the Dataset

**FIGURE 1:**
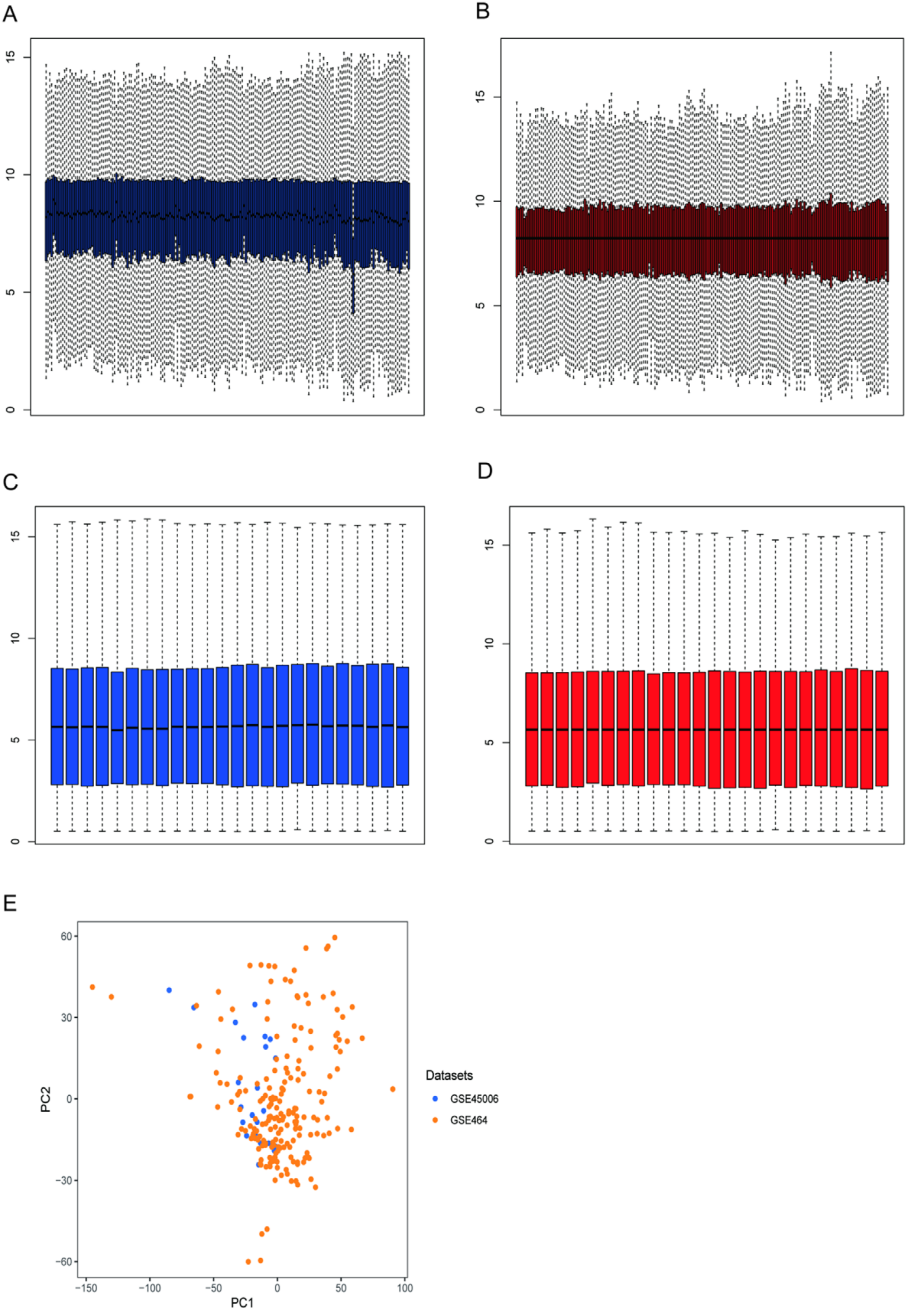
Normalisation and batch correction of the datasets. (A) and (B) are boxplots of samples in GSE464 before and after correction, respectively. (C) and (D) are boxplots of samples in GSE45006 before and after correction, respectively. (E) is PCA batch correction on the combined data.

We presented boxplots of the two datasets, GSE464 and GSE45006, before and after correction and the scatter plots of the PCA batch correction after merging in Figure 1. After normalisation, the gene expression values of each sample were at the same level, which indicates that gene expression values of each sample were comparable. After the yellow and blue points were corrected in time by PCA batches, the influence of two batches of dataset was eliminated and the two datasets were comparable.

### 3.2 Identification and Enrichment Analysis of DEGs

**FIGURE 2:**
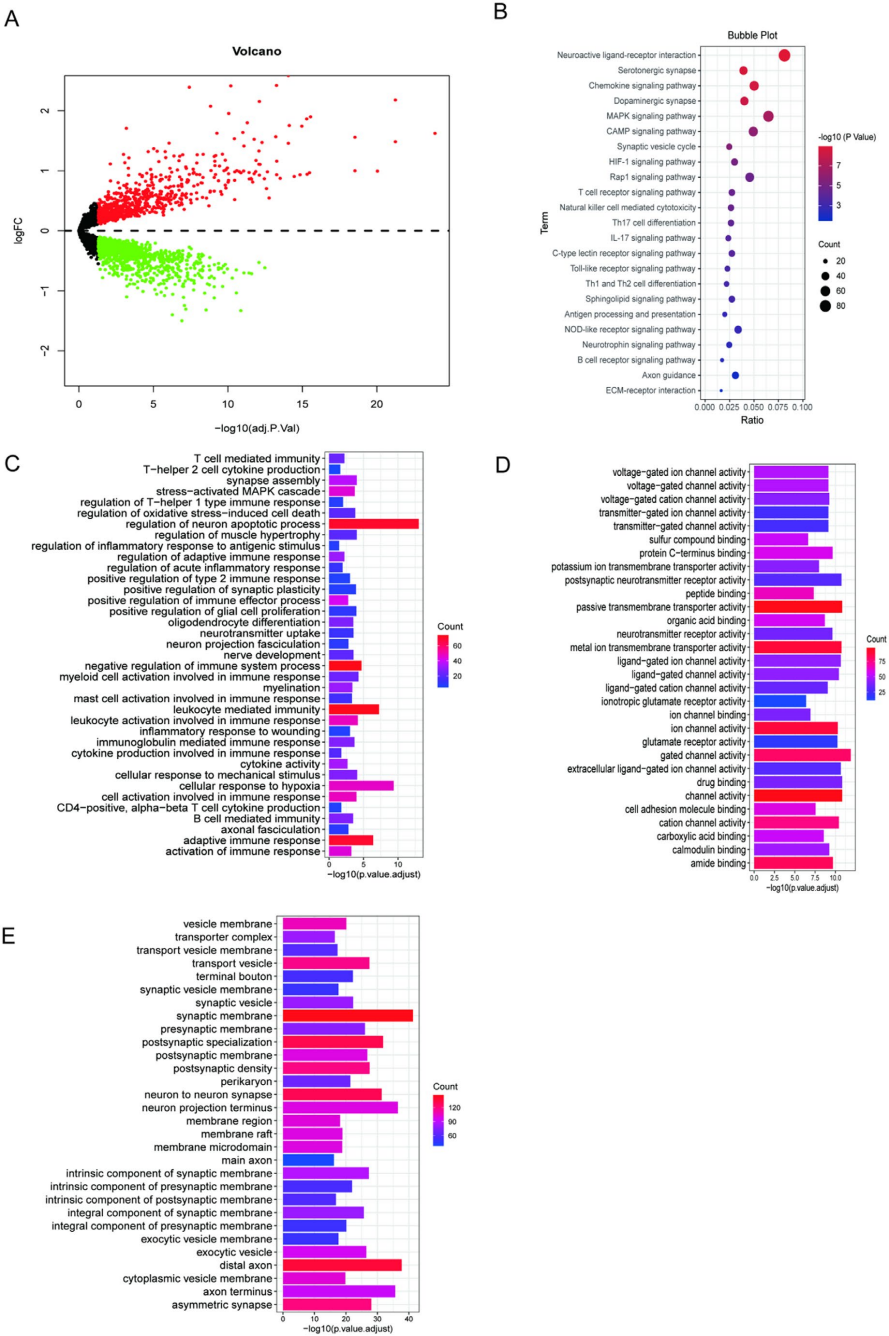
Analysis of differences and changes in biological function before and after SCI. (A) is the volcano plot obtained from the difference analysis before and after SCI. (B) is the result of the KEGG enrichment analysis of DEGs. (C–E) are results from three categories of BP, MF, and CC in GO enrichment analysis of differentially expressed genes.

In this section, we combined day 1, 14 and week > 4 samples as the injury group and compared with the control group (FDR < 0.05). Then we obtained differentially expressed genes (DEGs) of the SCI group and control group, and performed GO and KEGG enrichment analysis (Figure 2). Volcano plots represented DEGs with 293 up‐regulated genes (red) and 249 down‐regulated genes (green; Figure 2A), including those related to inflammation, regeneration, ischaemia, etc. The results of KEGG enrichment analysis (Figure 2B) and GO enrichment analysis including BP, MF and CC (Figure 2C–E) showed that immune‐related signalling pathways were significantly activated and more enriched after SCI, especially T cell‐related pathways. Although the above results indicated that immune‐related progression continued to occur after SCI, regardless of whether it promoted or inhibited repair, the immune responses at different time points after injury are not unchanging, so further exploration is needed.

### 3.3 Identification of Constant DEGs

**FIGURE 3:**
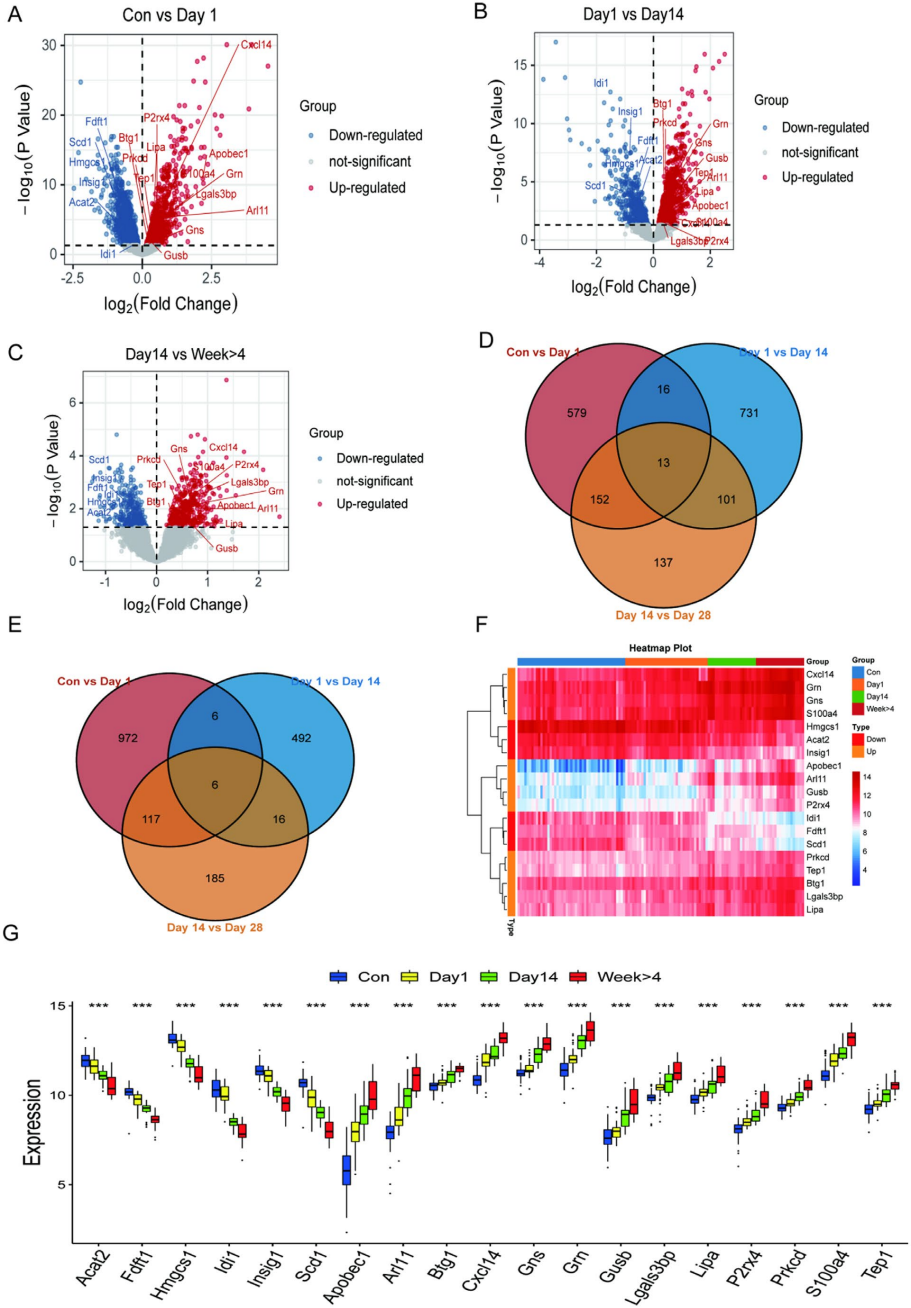
Identification of constant DEGs. (A–C) are the volcano plots obtained from pairwise difference analysis of the four data groups. (D) and (E) are Venn plots of constant up‐regulated and constant down‐regulated genes after pairwise analysis of the four data sets, respectively. (F) and (G) are heatmaps and boxplots of the expression differences of the 19 identified constant DEGs before and after SCI disease, respectively.

To explore the differences in immune responses at different time points after injury, we further selected three DEGs that were obtained by differential analysis of Day 1 versus Con, Day 14 versus Day 1, and Day 28 versus Day 14 to analyse. The three volcano plots (Figure 3A–C) intuitively showed the DEGs in the three groups (Day 1 vs. Con; Day 14 vs. Day 1; Day 28 vs. Day 14), and we found there were some genes that were always consistently expressed at different time points after injury. As seen from the two Venn plots (Figure 3D,E), there was a total of 19 DEGs, including 13 DEGs that were consistently up‐regulated, for example, the GRN gene, and 6 consistently down‐regulated as time went on after SCI. Then we analysed qualitatively and quantitatively the constant expression differences of these 19 genes before and after SCI by differential expression heatmap and differential boxplot (Figure 3F,G). These results suggested that these 19 DEGs might play essential regulatory roles after SCI and were expressed steadily.

### 3.4 Correlation Between Constant DEGs and Changes in Immune Microenvironment After SCI

**FIGURE 4:**
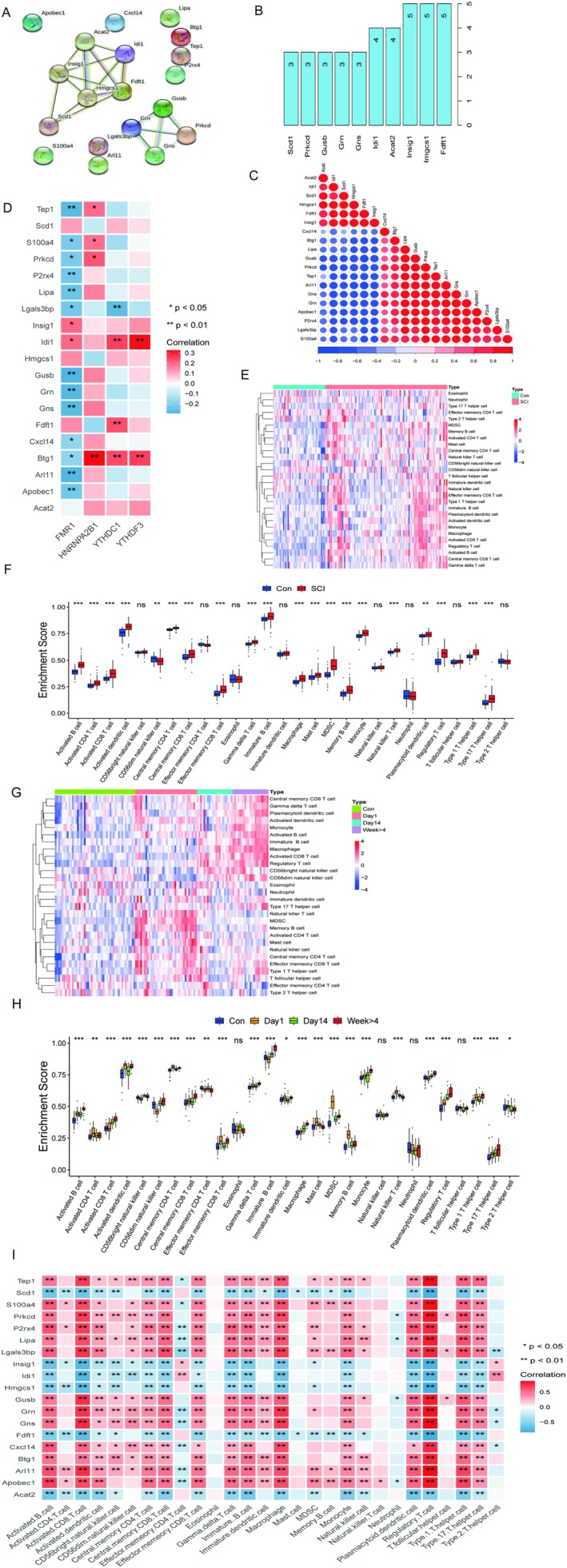
Correlation between constant DEGs and changes in immune microenvironment after SCI. (A) is the PPI protein interaction network drawn by constant DEGs. (B) is the number of nodes connected between each protein and other proteins. (C) is the heatmap of the correlation of constant genes on mRNA expression, blue indicates a negative correlation, and red suggests a positive correlation. (D) is the heatmap of the correlation between the expression of 19 genes and m6A methylation regulators. (E) is the heatmap of the difference in immune cells between the Con and SCI groups. (F) is the boxplot of the difference in immune cells between the Con and SCI groups. (G) is the heatmap of the differential expression of immune cells grouped by time. (H) is the boxplot of the differential expression of immune cells grouped by time. (I) is the heatmap of correlation between the expression of 19 genes and immune cells.

We analysed the PPI protein interaction network revealing significant connections of 19 constant DEGs at the protein level (Figure 4A). The number of PPI nodes indicated the number of nodes connected between each protein and the other 18 proteins (Figure 4B). Next, we conducted the correlation of mRNA expression of the 19 genes showing that there were significant correlations in mRNA expression (Figure 4C). Many studies have shown N6‐methyladenosine (m6A) methylation is very significant to modify messenger RNA (mRNA), which affected and regulated many biological processes and plays an essential role in mRNA splicing, export, translation and other processes. So, we needed to analyse the correlation among the expression of 19 genes and m6A methylation regulators (asterisks indicate correlation), and the result showed that the expression of Btg1 and Idi1 was more relational to m6A methylation (Figure 4D). According to Result 3.2, the immune‐related signalling pathways were significantly activated and more enriched after SCI. We used a qualitative difference heatmap (Figure 4E) and a quantitative difference boxplot (Figure 4F) to reveal the changes in the immune microenvironment after SCI, respectively. And we found that there were abundant immune cells activated after SCI. Specifically, we measured the content of 28 immune cells using the ssGSEA algorithm and found significant differences in immune cell content between the SCI and the Con groups. Subsequently, to analyse the content of immune cells at different time points after SCI, we used a differential heatmap (Figure 4G) and differential volcano map (Figure 4H) to find the changes in immune cells with the time of SCI, showing that immature B cell and regulatory T cell were continuously activated, dendritic cells and myeloid‐derived suppressor cells (MDSCs) were activated at 1 day after injury and decreased as the time went on. Considering that the changes in the immune microenvironment may play a crucial role after SCI, we did a correlation analysis between 19 DEGs and 28 immune cells. The results showed that constant DEGs significantly correlated with immune microenvironment cells (Figure 4I).

### 3.5 Correlation Between Constant DEGs and Changes in Immune Microenvironment After SCI

**FIGURE 5:**
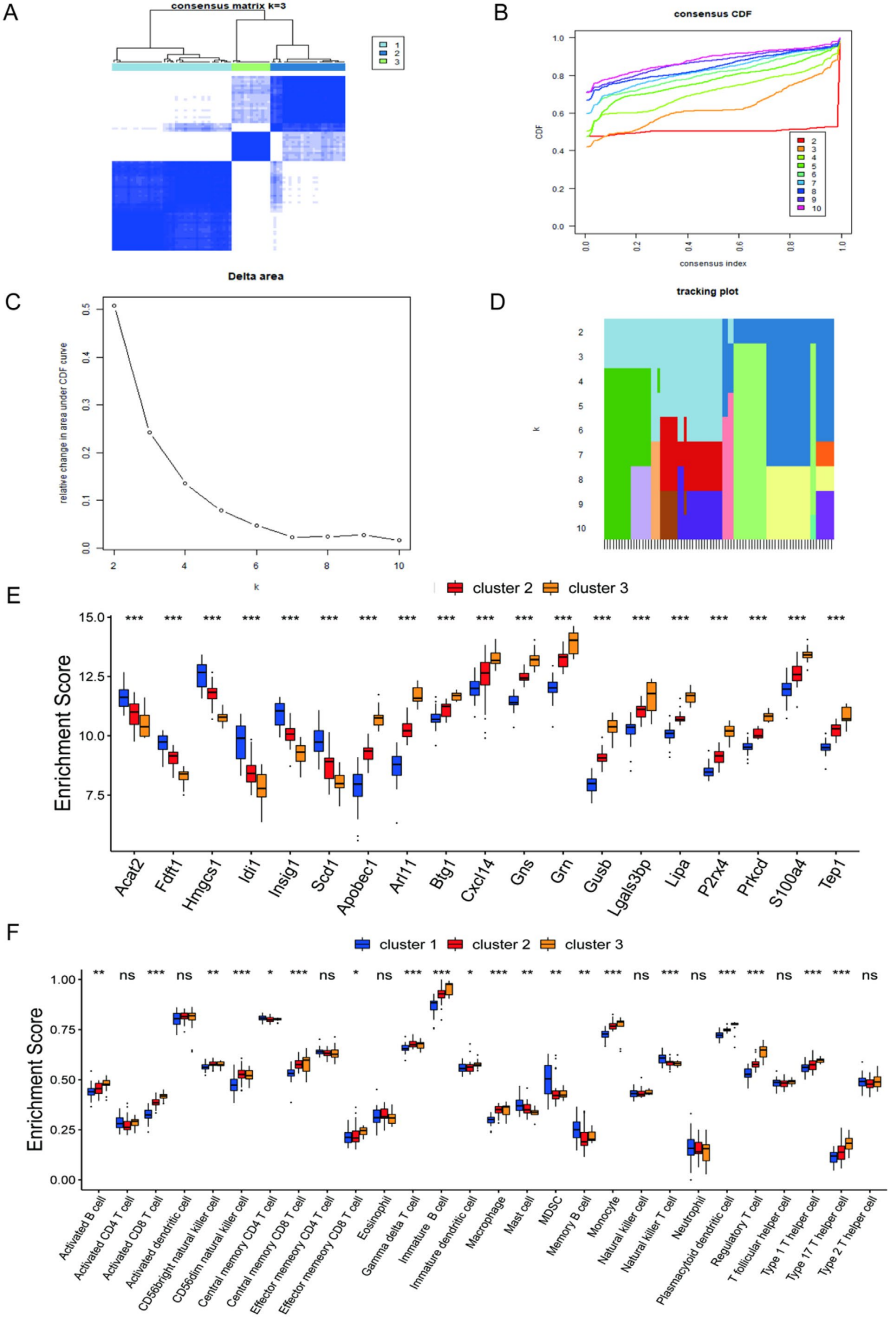
Identification of SCI molecular subtypes based on 19 constant genes. (A) is the consistency matrix heatmap for k=3. (B) is the line graph that uses the cumulative density function (CDF) to measure the stability of the cluster when *k* takes 2–10. (C) is the relative change in the area under the curve of the CDF as the number of clusters increases. The columns of (D) are the samples, and the rows are the values of the number of different clusters. Colours indicate categories in the agreement matrix. (E) is a boxplot of the expression of 19 constant DEGs in the three isoforms. (F) is the boxplot plotted by the expression of 28 immune cells in the three subtypes.

We used a consensus clustering algorithm to identify three different molecular subtypes of SCI based on the expression of 19 constant DEGs. We confirmed that better clustering results could be obtained by setting the number of subtypes to 3 (Figure 5A–D). Then we found that 19 constant DEGs and 28 immune cells significantly differed in the three groups of molecular subtypes (Figure 5E,F).

### 3.6 Identification of Potential Immunobiological Markers of SCI Severity

**FIGURE 6:**
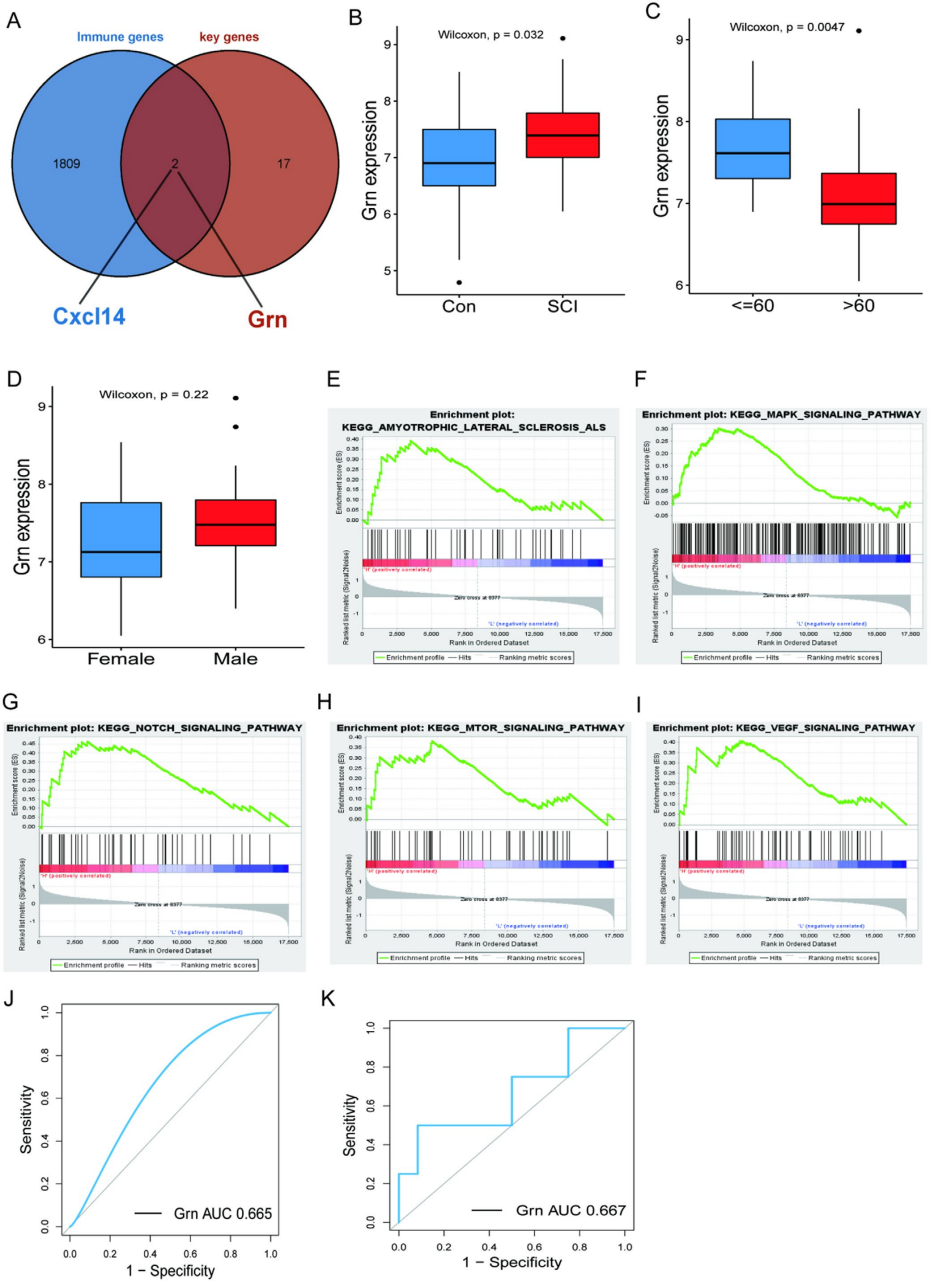
GRN is a potential immunobiological marker for monitoring the severity of spinal cord injury. (A) is a Venn diagram of the intersection of 19 constant DEGs and immune genes. (B) is the boxplot of GRN expression between SCI patients and normal samples. (C) is age ≤ 60 and > 60 boxplot of GRN expression in SCI patients. (D) is the boxplot of GRN expression in male and female patients with SCI. (E–I) is the possible regulatory pathway in which the GRN may be involved using GSEA enrichment analysis. (J) is the ROC curve of GRN pairs to diagnose whether SCI occurred or not. (K) is the ROC curve of GRN for predicting the degree of injury in SCI patients.

Considering the critical role of the immune microenvironment in SCI, we overlapped 19 DEGs with 1811 immune genes and obtained 2 immune genes, including GRN and CXCL14 (Figure 6A). Considering that the differential fold expression of GRN after SCI was significantly higher than that of cxcl14, we used GRN as a backup biomarker for SCI. Next, we used human peripheral blood transcriptome data of SCI to verify whether GRN could be used as a potential biomarker for monitoring the severity of SCI. The GSE151371 dataset showed that the expression of GRN in the peripheral blood of SCI patients was significantly higher than that of normal samples (Figure 6B), and its expression was correlated with the age of patients (Figure 6C) and low expression in elderly patients, but not with gender (Figure 6D). We further analysed the possible regulatory pathways in which GRN was involved using GSEA enrichment analysis and found GRN may be involved in MARK, NOTCH, and mTOR signalling pathways (Figure 6E–I). We additionally measured the efficacy of GRN as a haematological marker to diagnose the occurrence of SCI (Figure 6J) and to predict the degree of injury (Figure 6K). Among them, the AUC for predicting the occurrence of SCI was 0.665. The AUC for predicting the degree of injury was 0.667. This means that GRN has specific predictive efficacy. In future studies, we will try to expand the sample size to investigate the diagnostic significance of GRN further.

### 3.7 Identification of Candidate Drug That Is Related With GRN Gene in SCI

The exploration of protein‐drug interaction was a crucial step in the translation of prediction results of potential disease targets. In the aspects of GRN genes as potential drug targets in SCI, transcriptomic signatures of the DSigDB database based on Enrichr provide us with multiple candidate drug screening results, and quinpirole, chloramphenicol, emetine, epitiostanol, felodipine, and pronetalol were identified with more significance. These drug compounds may play a significant role in intervening in the GRN gene to repair SCI. Table 1 points out information on the significance result, chemical formula, and structure of prediction result.

**TABLE 1:** Identification of candidate drug related with GRN.

Drug name	*p*‐value	*S*‐value	Chemical formula	Structure
Chloramphenicol	0.0006	−5.5055	C_11_H_12_Cl_2_N_2_O_5_	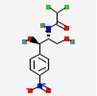
Emetine	0.00034	−7.1465	C_29_H_40_N_2_O_4_	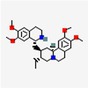
Epitiostanol	0.00199	−4.8713	C_19_H_30_OS	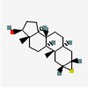
Felodipine	0.00228	−5.8287	C_18_H_19_C_l2_NO_4_	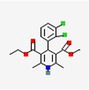
Pronetalol	0.00251	−5.3358	C_15_H_19_NO	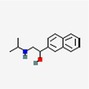
Quinpirole	0.00056	−4.9209	C_13_H_21_N_3_	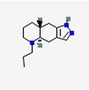

### 3.8 Validation of Hub PCD Genes and the Effect of Quinpirole Treatment in Rat SCI

**FIGURE 7:**
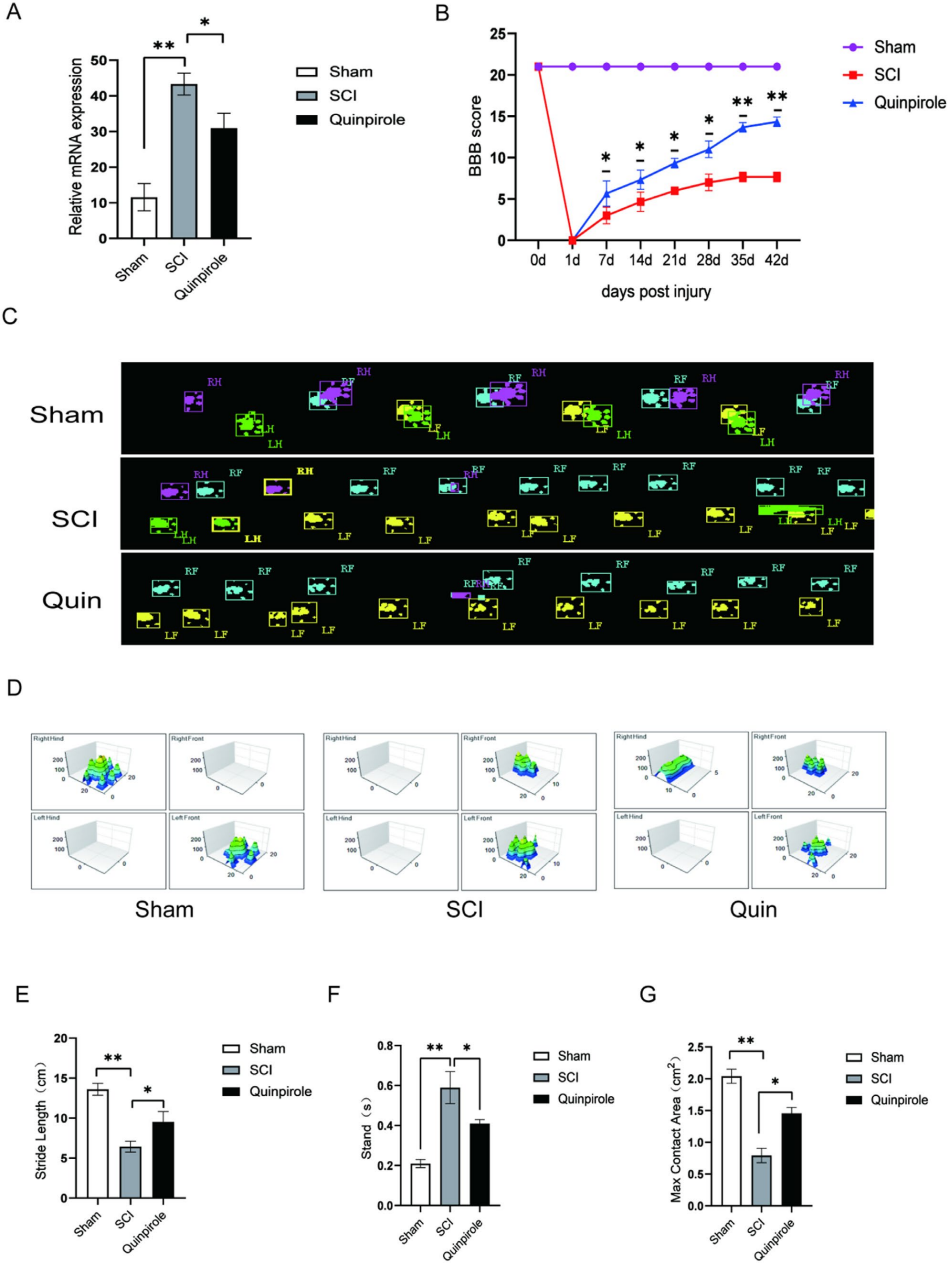
Expression changes of hub gene and effect of Quinpirole on functional recovery. (A) mRNA expression level of GRN gene in Sham, SCI, and Quinpirole group. (B) Basso‐Beattie‐Bresnahan (BBB) were performed to assess lower limb motor function in three groups. (C–G) Catwalk XT and gait recording were performed to assess lower limb motor function. ***p < 0.001, **p < 0.01, *p < 0.05.

We collected the spinal cords of the Sham group, the SCI group, and the SCI + Quinpirole, and detected the expression of GRN genes using qPCR. Consistent with the results of analysis, the expression of the GRN gene was significantly up‐regulated in the SCI group compared with the Sham group, and after treatment, its expression level had been down‐regulated (Figure 7A).

To confirm the therapeutic effect of quinpirole after SCI, quinpirole treatment was administered directly 1 dpi at a dosage of 2 mg/kg/day for 5 days. Hindlimb movement was measured by the Basso‐Beattie‐Bresnahan (BBB) locomotor test. One day after injury, BBB scores of injured animals dropped to 0. With the passage of time, animals in the SCI group gradually developed ankle movements, including spasms, resulting in an average final score of less than 8. Quinpirole treatment resulted in a significant progressive recovery of locomotion, which began from the second week and the final score reached 14 (Figure 7B).

In addition, footprint analysis was used to test detailed locomotion with the Catwalk XT system. In the quinpirole treatment group, coordinated movement of fore‐nd hind‐limbs was observed; meanwhile, all rats in the SCI group failed to show any hind paw placement. Moreover, hindlimb base of support significantly increased following treatment (Figure 7C,D). Stride length decreased significantly in the forelimb after SCI; however, it recovered after treatment (Figure 7E). Also, functional restoration after quinpirole treatment allowed quantification of several parameters, such as the stand time and max contact area, which reached 60%–80% of the uninjured controls (Figure 7F,G).

**FIGURE 8:**
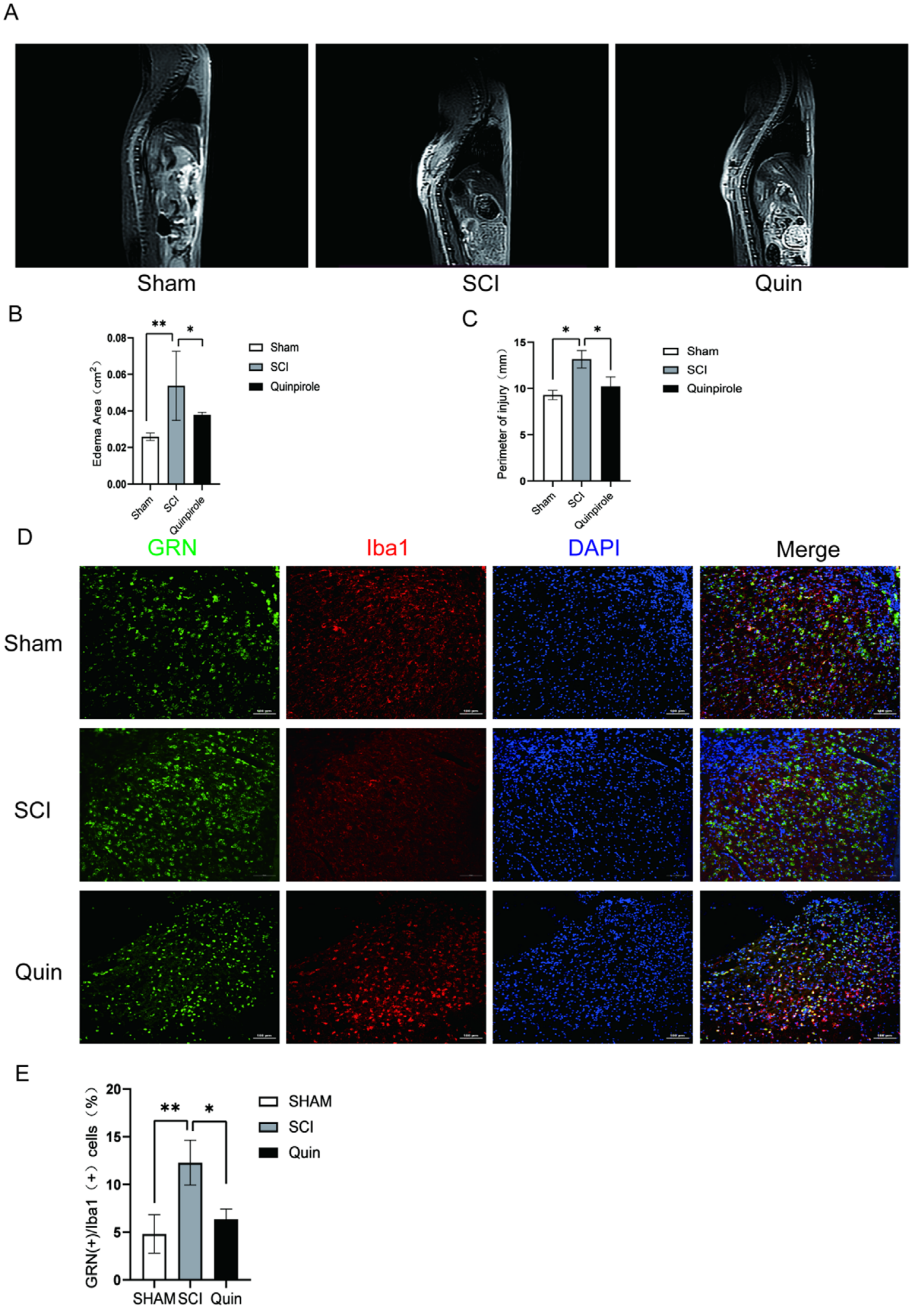
Effect of Quinpirole to functional recovery after SCI. (A–C) Magnetic Resonance Imaging (MRI) were performed to access degree of SCI. (D, E) Immunofluorescence staining with GRN (green) and Iba1 (red) were performed to detect the recovery treated with Quinpirole. *p < 0.05, **p < 0.01, ***p < 0.001.

To further confirm the therapeutic effect of quinpirole after SCI, we used Magnetic Resonance Imaging. Normal spinal cord was a columnar structure within the spinal canal and could clearly distinguish the cerebrospinal fluid in the spinal cord and subdural space. The spinal cord signal was uniform and slightly higher than the back muscle signal. In the SCI group, the skin and soft tissue of the back were significantly swollen, with unclear structure. Focal low signal shadows could be seen in the thoracic spinal cord, and intramedullary T2W1 high signal shadows accompanied by spinal cord thickening. However, in the quinpirole group, the swelling degree of the back skin and soft tissue had decreased, the signal of the thoracic spinal cord was irregular, and no obvious cystic changes had been observed. The T2W1 high signal shadow in the medulla had shrunk, and the degree of spinal cord swelling had decreased (Figure 8A–C). Then we used an immunofluorescence method to evaluate the effect of quinpirole on the expression of the GRN gene, showing that the expression of the GRN gene in microglia (the most important immune cells in the central nervous system) was a noticeable increase after SCI, but it decreased closer to the Sham group after treatment with quinpirole (Figure 8D,E).

## 4 Discussion

As a severe injury, SCI may lead to permanent sensory and motor function impairment. Previous studies confirmed that changes in m6A methylation regulators and the immune microenvironment play an essential role in the development of SCI. This paper obtained SCI and normal samples in GSE464 and GSE45006. The two data sets were combined after removing batch effects. Next, DEGs of SCI and normal samples were obtained by differential expression analysis. GO and KEGG enrichment analysis was performed for these DEGs. Most of the pathways involved in DEGs are closely related to the occurrence and progress of immunity and SCI. Specifically, based on the DEGs obtained from the difference analysis before and after SCI in Result 3.2, the relevant pathway information of their involvement was obtained by enrichment analysis in this paper. Among them, the relationship between some pathways and SCI has been confirmed by previous studies. For BP‐related pathways, Schwartz and Hauben [27] showed that traumatic injury to the CNS associated with SCI spontaneously elicits purposeful T cell‐mediated autoimmune responses. Bertels et al. [28] found that adult SCI prompts neurotransmitters to switch excitatory interneurons into inhibitory phenotypes, thereby promoting synapses inhibition in contact with motor neurons. Lin et al. [29] found that pycnogenol could achieve neuroprotection in rats with SCI by stabilising mitochondrial membrane potential. Na+/K+‐ATPase is a transmembrane ion pump. Its loss‐of‐function disease mutation G301Rα^++^_2__2 subtype can reduce the disease variables and improve the functional outcome after acute SCI in mice [30]. Guo et al. [31] treated mouse embryonic stem cell conditioned medium with myelin‐loaded macrophages as immunomodulatory therapy for inflammatory injury after SCI, which improved the exocytosis of internalised lipids, and the phagocytosis ability of apoptotic cells was normal. For CC‐related pathways, Deng et al. [32] tested two small peptides in neurite outgrowth assays and SCI models to examine the effect of these molecules on the inhibition of Nogo‐66/NgR signalling. Zheng et al. [33] found that the association between Postsynaptic density‐95 (PSD‐95) and neuronal nitric oxide synthase changed substantially after SCI. It also found that presynaptic glutamate N‐methyl‐D‐aspartate receptor (NMDA) receptors control nociceptive transmission at the spinal cord level in neuropathic pain [34]. Angela's experimental results showed that death‐inducing signalling complex (DISC) proteins were recruited to membrane rafts after SCI [35]. Shuhei established a method to efficiently induce M1 or M2 microglia by exposure to granulocyte‐macrophage colony‐stimulating factor (GM‐CSF) or interleukin (IL)‐4 and found that intramuscular injection of fluororuby dye showed restoration of retrograde axonal transport from the neuromuscular junction upstream of the injured spinal cord only in the M2 transplant group. This result suggests that M2 microglia may be a potential treatment for SCI after stimulation [36]. For MF‐related pathways, CPTX can interact with presynaptic neurotoxins and postsynaptic AMPA‐type ionic glutamate receptors and induce excitatory synapse formation in vitro and in vivo. CPTX can restore the synaptic function of SCI mice [37]. Alvarez et al. [38] found that a peptide amphiphilic supramolecular signal activated the transmembrane receptor β1‐integrin in a mouse model, which enhanced the movement of molecules within the scaffold fibrils and promoted the recovery of spinal cord injury. For the pathways associated with KEGG enrichment analysis, it has been shown that peripheral nerve injury produces neuropathic pain and memory deficits and induces long‐term potentiation at C‐fibre synapses in the spinal dorsal horn [39]. In addition, Sarah et al. [40] explored the addictive potential of morphine after SCI. Gutierrez et al. [41] found that the injury occurred in the puerperium of rats, which would cause oxytocin signalling in the central nervous system and make the hypersensitivity reaction caused by peripheral nerve injury quickly subside.

Next, we performed differential analysis and obtained three groups of DEGs for Day 1 versus Con, Day 14 versus Day 1, and Day 28 versus Day 14. Then the intersection of the three groups of DEGs was taken to obtain 19 constant DEGs. We conducted a correlation analysis between constant DEGs and m6A methylation regulators and SCI immune microenvironment changes, respectively, and found that they had a strong correlation. In addition, we performed biological significance analysis for 19 constant DEGs. Specifically, 19 constant DEGs were obtained in this paper according to the multi‐group difference analysis in Result 3.3. The close relationship between some DEGs and the occurrence and development of SCI has been confirmed. The experiments of Sebastian et al. [42] verified the strong up‐regulation of SCD1 at the mRNA and protein levels in regenerating neurons of the rat facial nucleus. Erin M Triplet et al. [43] found that functional recovery and neural repair signs were increased after the knockout of protease‐activated receptor 1 (PAR1) in SCI of female mice, and PAR1 differentially regulates HMGCS1. The ketogenic diet plays a protective role and promotes functional recovery after SCI. Zeng et al. [44] found significant differences in Fdft1 in rats fed a ketogenic diet. In a study on the molecular mechanism of SCI in rats, Chen et al. [45] found that the expression level of IDI1, a gene related to cholesterol metabolism, was negatively correlated with the duration of SCI. Shen et al. [46] established an SCI rat model and showed that miR‐34c could promote neuronal recovery in SCI rats by inhibiting CXCL14 expression and inactivating the JAK2/STAT3 pathway. Chen et al. [47] identified APOBEC1 as a key gene in contused SCI by analysing microarray data. In addition, GUSB [48], P2RX4 [49], and S100a4 [50] have also been associated with SCI.

Finally, this paper used a consensus clustering algorithm to classify SCI patients into three subtypes. Most of the 19 constant DEGs among different subtypes significantly differed with 28 immune cell contents. In addition, GRN is an intersection gene between constant DEGs and immune genes. We compared the differences in GRN expression by age, gender, and presence or absence of disease. Then in the external test set GSE151371, GRN was used to diagnose the occurrence of SCI (AUC = 0.665) and predict the degree of SCI patients (AUC = 0.667). In addition, the possible regulatory pathways involved in GRN were further analysed by GSEA enrichment analysis. We analysed the biological significance analysis of these regulatory pathways. Specifically, this paper used GSEA enrichment analysis in Result 3.6 to identify regulatory pathways in which GRN, an intersection gene of immune genes and constant DEGs, might be involved. Progranulin, which is encoded by the GRN gene, is a family of secreted, glycosylated peptides, which play great roles in regulating lysosomal function and as a growth factor involved in inflammation, wound healing, cell proliferation, and so on [51]. It has been found that GRN expression was up‐regulated after spinal cord injury [52]; however, the potential relationship between GRN and the immune microenvironment has not been clarified clearly. A TCGA analysis found GRN as a prognostic biomarker in glioma which might be related to immune infiltration [53]. Progranulin deficiency could lead to neurodegeneration which is regulated by complement activation and microglia‐mediated synaptic pruning [54]. Interestingly, in renal ischemia/reperfusion injury, whose pathological mechanism is similar to spinal cord injury, GRN was found significantly reduced, which is opposite to spinal cord injury [55]. In addition, GRN deficiency could significantly aggravate renal injury and increase the infiltration of neutrophils and macrophages in renal tubulointerstitial. The administration of GRN could promote recovery from renal ischemia/reperfusion injury. GRN augmentation has been considered for the treatment of a series of neurodegenerative diseases including Parkinson's disease, Alzheimer's disease, and so on. However, over‐expression of GRN in the brain is deleterious and will lead to severe neurodegeneration, which might be caused by T cell‐mediated inflammatory response [56]. Some pathways have been confirmed to be associated with the onset and progression of SCI. Eva Sykova et al. identified autologous and allogeneic mesenchymal stem cells (MSCs) as potential treatments for SCI and amyotrophic lateral sclerosis (ALS) [57]. J Ye et al. explored the effect of neurotrophin‐3 (NT‐3) on SCI repair through the mitogen‐activated protein kinase (MAPK) signalling pathway. They found that NT‐3 could inhibit the MAPK signalling pathway, thereby promoting SCI repair [58]. Cheng et al. found that early surgical decompression could partially promote the recovery of motor function, possibly through the activation of the Notch‐1 signalling pathway after spinal cord compression injury [59]. In addition, Ding et al. experimentally found that the mTOR pathway is a potential therapeutic target for SCI [60]. Wang et al. found that VEGF inhibits inflammation in SCI by activating autophagy [61]. Future research needs to further explore the specific mechanism of the GRN gene in nervous system diseases, in order to provide new targets and strategies for the prevention and treatment of related diseases.

In order to achieve better clinical conversion and treatment, the protein‐drug interaction was a crucial step and predicted results of potential disease targets. We found that quinpirole can improve the microenvironment after spinal cord injury by targeting the GRN gene and lowering its level to repair spinal cord injury. Quinpirole is a selective dopamine D2/D3 receptor agonist, which is mainly used to study the function of the dopamine receptor and related diseases. The dopamine system interacts with other neurotransmitter systems, such as GABAergic and glutamatergic systems. Quinpirole may indirectly affect these systems by activating D2/D3 receptors, thus producing more extensive effects in the nervous system. For example, studies on the effects of dopamine agonists Ropinirole and Quinpirole on the locomotion and anti‐anxiety behaviour of zebrafish larvae showed that Ropinirole upregulated the transcripts related to GABA and glutamate systems, while Quinpirole did not significantly change the abundance of these transcripts. Considering the application of Quinpirole in the clinical treatment of Parkinson's disease (PD), epilepsy and neuroinflammation, the safety of Quinpirole in the treatment of spinal cord injury will be guaranteed.

In this study, we have to admit that there are still some limitations. As we all know, transcriptome technology needs a large number of samples for sequencing. For the experiments with small sample sizes, the accurate analysis results may not be obtained. There were two datasets used in this study; although the data sample size was not large enough, we might obtain scientific results through rigorous bioinformatics analysis. On the other hand, we chose the rat spinal cord contusion model, which is similar to the pathological mechanism of human spinal cord injury. Only female Wistar rats were used in this study; there is no relevant report on whether the difference in gender or hormone levels will affect the effect of Quinpirole. It is still not clear whether the effect of Quinpirole on the spinal cord transection injury model or spinal cord clamp injury model is different. By the way, the application of single‐cell sequencing technology can also effectively improve the heterogeneity from other cell sources. Quinpirole has been used in the treatment of many human nervous system diseases, including Parkinson's Disease (PD), epilepsy, and so on. Based on the results of this study, we believe that Quinpirole has good transformation potential in the clinical treatment of spinal cord injury.

## 5 Conclusion

In conclusion, this study identified 19 DEGs associated with SCI through multiple bioinformatics analysis methods. The correlation between these DEGs and m6A regulatory factors and immune cell content was analysed in detail. In addition, three subtypes of SCI were identified by consensus clustering. The three subtypes were significantly different in terms of constant gene expression. Further analysis identified the significance of GRN in diagnosing SCI and predicting the degree of SCI injury, and speculated that GRN was a potential immunobiological marker. Finally, quinpirole is an effective drug to target GRN to repair SCI.

## Author Contributions

**Han Ding:** writing – original draft (equal), writing – review and editing (lead). **Lei Feng:** writing – original draft (equal), writing – review and editing (equal). **Jianping Zhang:** writing – original draft (equal), writing – review and editing (equal). **Tuo Fang:** data curation (equal), writing – original draft (equal), writing – review and editing (equal). **Jun Shang:** data curation (equal), formal analysis (equal). **Ke Fang:** methodology (supporting). **Shiqing Feng:** project administration (lead).

## Ethics Statement

Animal study was reviewed and approved by the Ethics Committee of the Institute of Tianjin Medical University General Hospital (approval number: IRB2022‐DW‐45) and performed according to the national guidelines for laboratory animal use and care.

## Conflicts of Interest

The authors declare no conflicts of interest.

## Data Availability

The data that support the findings of this study are available in GEO database (https://www.ncbi.nlm.nih.gov/geo/).
